# Genetic polymorphisms and asthma: findings from a case–control study in the Madeira island population

**DOI:** 10.1186/0717-6287-47-40

**Published:** 2014-09-04

**Authors:** Anabela Gonçalves Berenguer, Ana Teresa Fernandes, Susana Oliveira, Mariana Rodrigues, Pedro Ornelas, Diogo Romeira, Tânia Serrão, Alexandra Rosa, Rita Câmara

**Affiliations:** Human Genetics Laboratory, University of Madeira, 9000-390 Funchal, Portugal; Immunoalergology Unit, Dr. Nélio Mendonça Hospital, SESARAM, E.P.E, Funchal, Portugal; Unit of Statistics, Dr. Nélio Mendonça Hospital, SESARAM, E.P.E, Funchal, Portugal; Medical Sciences Unit, Center of Life Sciences, University of Madeira, 9000-390 Funchal, Portugal; Department of Computer Science and Engineering, University of Oulu, PL 4500, 90014 Oulu, Finland

**Keywords:** Asthma, Madeira, SNPs, Susceptibility, Severity

## Abstract

**Background:**

Asthma is a complex disease influenced by multiple genetic and environmental factors. While Madeira has the highest prevalence of asthma in Portugal (14.6%), the effect of both genetic and environmental factors in this population has never been assessed. We categorized 98 asthma patients according to the Global Initiative for Asthma (GINA) guidelines, established their sensitization profile, and measured their forced expiratory volume in 1 second (FEV_1_) and forced vital capacity (FVC) indexes. Selected single nucleotide polymorphisms (SNPs) were analysed as potential markers for asthma susceptibility and severity in the interleukin 4 (*IL4*), interleukin 13 (*IL13*), beta-2-adrenergic receptor (*ADRB2*), a disintegrin and metalloprotease 33 (*ADAM33*), gasdermin-like (*GSDML*) and the signal transducer and activator of transcription 6 (*STAT6*) genes comparatively to a population reference set.

**Results:**

Although mites are the major source of allergic sensitization, no significant difference was found amongst asthma severity categories. IL4-590*CT/TT and IL4-RP2*253183/183183 were found to predict the risk (2-fold) and severity (3 to 4-fold) of asthma and were associated with a lower FEV_1_ index. ADRB2-c.16*AG is a risk factor (3.5-fold), while genotype GSDML-236*TT was protective (4-fold) for moderate-severe asthma. ADAM33-V4*C was associated to asthma and mild asthma by the transmission disequilibrium test (TDT). Finally, ADAM33-V4*CC and STAT6-21*TT were associated with higher sensitization (mean wheal size ≥10 mm) to house dust (1.4-fold) and storage mite (7.8-fold).

**Conclusion:**

In Madeira, *IL4-590C/T*, *IL4-RP2 253/183*, *GSDML-236C/T* and *ADAM33-V4C/G* SNPs are important risk factors for asthma susceptibility and severity, with implications for asthma healthcare management.

**Electronic supplementary material:**

The online version of this article (doi:10.1186/0717-6287-47-40) contains supplementary material, which is available to authorized users.

## Background

Asthma is a chronic airway inflammation, characterized by variable airway obstruction and reduced lung function, and leading to wheezing and shortness of breath [1]. It is described as a complex disease arising from the contribution of multiple genetic and environmental factors [2, 3], affecting more than 300 million people worldwide, with increased prevalence in developed societies [4].

In Portugal, 7.83% of the population is affected by clinical asthma and 8.72% by wheezing symptoms [5]. Within Portugal, the island of Madeira has a high prevalence of the disease, as active asthma (presence of symptoms during the last 12 months) affects 14.6%, and atopy (positive reaction to skin prick tests (SPT) to common aeroallergens and the presence of specific IgE) affects 54% of the population [6].

Given the high prevalence of asthma in the Madeira population [6], determining the population’s genetic background is an essential first step towards understanding the mechanisms of the disease. In previous work [7] we showed that both *IL4-590* and *IL4-RP2* are useful genetic markers to detect asthma predisposition in the Madeira population. Investigating a larger number of genes is important because many genes with small effects, rather than just a few with strong effects, contribute to the disease risk [8]. A useful clustering of asthma susceptibility genes has been proposed [9]: i) triggers of the immune response (such as *CD14, IL10*, *STAT3*, MHC class II molecules*)*; ii) regulators of the T helper 2 (Th2) differentiation (*IL12B*, *IL4*, *IL13*, *STAT6, IL4RA)*; iii) associated with epithelial biology (*CCL5*, *FLG*, *SPINK5, GSDML*) and iv) linked to lung function, airway remodeling and asthma severity (*ADRB2, ADAM33*, *DPP10*, *PHF11).*

Here we extend our analysis in four important ways, to consider i) 6 additional polymorphisms, namely *IL13-c.144 G/A, ADRB2-c.16 A/G, ADAM33-V4 C/G, ADAM33-S1 c.710 G/A, GSDML-236 C/T* and *STAT6-21 C/T* (guided by a literature review summarized in Table 1), ii) the asthma persistence and severity classes according to GINA guidelines, iii) the most common allergens in the island and iv) the FEV_1_/FVC lung function indexes. Therefore, this work contributes to the analysis of the association of the above polymorphisms to asthma susceptibility and severity, FEV_1_/FVC indexes and sensitization profiles, in the Madeira population.

**Table 1:** **Selected studies previously reporting association of the studied SNPs and asthma**

Gene	Polymorphism	dbSNP rs ref (NCBI)	Chromosome location	Alleles (ancestral:derived)	References
*IL13*	*c.144*Gln/Arg	rs20541	5q31 exon 4	G > A	[10–16]
*IL4*	*590*	rs2243250	5q31 promoter	C > T	[17–23]
*IL4*	*RP2*	-	5q31 intron 2	253 > 183(del)	[24]
*ADRB2*	*c.16*Arg/Gly	rs1042713	5q31 exon	A > G	[25, 26]
*ADAM33*	*V4*	rs2787094	20p13 3’UTR	C > G	[27–30]
*ADAM33*	*S1 c.710*Val/Ile	rs3918396	20p13 exon 19	G > A	[27]
*GSDML*	*236*	rs7216389	17q21 intron 1	C > T	[31–39]
*STAT6*	*21*	rs324011	12q13-24 intron 2	C > T	[19, 40–44]

## Results

### Demographic and clinical characterization of the asthmatics

The demographic and clinical characterization of the asthmatic population analysed in this study are shown in Table 2. The patients’ mean age was 13.6 ± 4.3 years old, with 59.2% males. None of the asthma persistence or severity phenotypes was found sub-structured by the male-to-female ratio. The FEV_1_ and FVC indexes were significantly different between mild persistent and moderate-severe persistent asthma (Mean FEV_1_ t = 2.732, p-value = 0.008; FVC Mann–Whitney U = 456.0, p-value = 0.025).

**Table 2:** **Clinical characterization of the asthmatics study population**

	Study sample set					
	Overall asthma	Intermittent asthma	Persistent asthma	Mild	Moderate-severe	p-value
Sample size (n,%)	98 (100.0)	24 (24.5)	74 (75.5)	44 (59.9)	30 (40.5)	
Male/Female (%)	59.2/40.8	62.5/37.5	58.1/41.9	54.5/45.5	63.3/36.7	
Mean age (years + -s.d.)	13.6 + -4.3	14.1 + -3.5	13.4 + - 4.5	14.3 + -4.6	12.0 + - 4.1	
Mean FEV_1_ (%)	96.9 + -14.7	98.5 + -16.3	96.4 + -14.2	**99.9 + -10.7**	**91.2 + - 17.1**	**0.008**
Mean FVC (%)	95.0 + -12.8	97.4 + -12.1	94.3 + -13.0	**97.3 + -9.9**	**89.8 + -15.6**	**0.025**

Asthma classification according to GINA [75].

A Shapiro-Wilk test showed that FEV_1_ is normally distributed (p > 0.05) while FVC is not (p = 0.008). Thus, a t-test was used for the analysis of FEV_1_ while a Mann–Whitney test was used for FVC. Statistically significant p-values were found for comparisons between mild persistent and moderate-severe persistent asthma, denoted in bold.

Although persistent asthmatics were found more frequently sensitized to both cat and dog allergen than intermittent asthmatics (Additional file 1) no significant differences were observed for SPT positivity or degree of sensitization to the most common allergens in the island, namely dust and storage mites mix fungi, indoor fungi, cat or dog.

### SNP association to asthma risk and severity

The genotypic and allelic frequencies of each SNP among asthma subgroups and population reference set are described in Table 3 and Additional file 2, respectively. The genotypic distribution of SNPs in controls reflects previously published data on other European and Mediterranean populations [45, 46]. All SNP polymorphisms were found to be in HWE, except for *STAT6-21 locus* in mild persistent asthma (p-value = 0.038). Nevertheless, this SNP was considered in the analysis under a more stringent p-value cut-off of 0.01, since a slight deviation from HWE is allowed for SNPs linked to disease status [47].

**Table 3:** **SNP genotypic frequencies for the Madeira reference set and asthmatics study population**

SNP genotypes	Study sample set					
	Madeira reference set	Overall asthma	Intermittent asthma	Persistent asthma	Mild	Moderate-severe
	105^a^	98	24	74	44	30
*IL4-590 C/T*						
CC	0.800	0.643	0.708	0.622	0.705	0.500
CT	0.191	0.327	0.250	0.351	0.295	0.433
TT	0.009	0.031	0.042	0.027	0	0.067
**p-value**		**0.009**		**0.006**		**0.002**
**OR (95%CI)**		**2.222 (1.191-4.146)**		**2.433 (1.264-4.717)**		**4.000 (1.701-9.434)**
*IL4-RP2 253/183*						
253253	0.818	0.694	0.792	0.662	0.750	0.533
183253	0.155	0.276	0.167	0.311	0.250	0.400
183183	0.027	0.031	0.042	0.027	0	0.067
**p-value**		**0.027**		**0.013**		**0.002**
**OR (95%CI)**		**1.984 (1.040 -3.788)**		**2.296 (1.159 -4.547)**		**3.938 (1.657- 9.357)**
*IL13-c.144 G/A*						
GG	0.676	0.704	0.625	0.730	0.773	0.667
GA	0.305	0.267	0.333	0.257	0.205	0.333
AA	0.019	0.020	0.042	0.014	0.023	0
*ADRB2-c.16 A/G*						
AA	0.181	0.194	0.250	0.176	0.250	0.067
AG	0.457	0.480	0.458	0.486	0.364	0.667
GG	0.362	0.327	0.292	0.338	0.386	0.267
**p-value**						0.010*
**OR (95%CI)**						**3.500 (1.318-9.293)**
*ADAM33-V4 C/G*						
GG	0.029	0.010	0	0.014	0.023	0
CG	0.209	0.235	0.292	0.216	0.182	0.267
CC	0.762	0.755	0.708	0.770	0.795	0.733
*ADAM33-S1c.710*						
*G/A* GG	0.905	0.929	0.917	0.932	0.932	0.933
GA	0.095	0.071	0.083	0.068	0.068	0.067
AA	0	0	0	0	0	0
*GSDML-236 C/T*						
CC	0.171	0.163	0.167	0.162	0.136	0.200
CT	0.467	0.469	0.500	0.459	0.364	0.600
TT	0.362	0.367	0.333	0.378	0.500	0.200
**p-value**						0.008**
**OR (95%CI)**						**0.250 (0.086-0.730)**
*STAT6-21 C/T* ^b^						
CC	0.428	0.449	0.417	0.459	0.523	0.367
CT	0.486	0.418	0.458	0.405	0.295	0.567
TT	0.086	0.133	0.125	0.135	0.182	0.067

a) Except for *IL4-590* and *IL4-RP2* (n = 110);

b) Polymorphism not in HWE for mild asthma (p-value = 0.038).

χ^2^ (or Fisher’s exact test when appropriate) significant p-values and OR (95%CI), using the Madeira reference set as a control, are shown in bold (dominant model).

* Heterozygote model, significant p-values and OR (95%CI) for comparison between mild persistent and moderate-severe persistent; Dominant model, Fisher’s exact test p-value = 0.038, OR (95%CI) 4.667 (0.953-22.851); ** Recessive model, significant p-values and OR (95%CI) for comparison between mild persistent and moderate-severe persistent; Heterozygote model: Fisher’s exact test p-value = 0.039, OR 2.625 (1.011-6.817).

Table 3 shows significant effects for the genotypes IL4-590*CT/TT, IL4-RP2*183253/*183183*, ADRB2-c.16*AG/AA, and GSDML-236*TT. Specifically, the IL4-590*CT/TT genotypes were significantly more frequent in asthma patients (35.8% *vs.* 20.0%, χ^2^ p-value = 0.009; OR 2.222, 95% CI 1.191-4.146), persistent asthma (37.8% *vs.* 20.0%; χ^2^ p-value = 0.006; OR 2.433, 95% CI 1.264-4.717), and moderate-severe asthma *vs.* the reference set (20% *vs.* 50.0%; χ^2^ p-value = 0.002; OR 4.000, 95% CI 1.701-9.434). Genotypes IL4-RP2*183253/*183183* were significantly more frequent in asthma patients (30.7% *vs.* 18.2%; χ^2^ p-value = 0.027; OR 1.984, 95% CI 1.040-3.788), in persistent asthma (33.8% *vs.* 18.2%; χ^2^ p-value = 0.013; OR 2.296, 95% CI 1.159-4.547), and in the moderate-severe asthma *vs.* the reference set (46.7% *vs.* 18.2%; χ^2^ p-value = 0.002; OR 3.938, 95% CI 1.657-9.357). ADRB2-c.16*AG was significantly more frequent in moderate-severe asthma *vs.* mild asthma (66.7% *vs.* 36.4%, p-value = 0.010, OR 3.500, 95% CI 1.318-9.293) while GSDML-236*TT displays a significant opposite trend (20.0% and 50.0%, respectively in moderate-severe and mild asthma; χ^2^ p-value = 0.008, 0R 0.250 95% CI 0.086-0.730).

The results described above are corroborated by the allelic counts (Additional file 2) of IL4-590*T, which are significantly higher in asthma patients (19.4%; χ^2^ p-value = 0.010, OR 2.060, 95% CI 1.178-3.601), in persistent asthma (20.3% χ^2^ p-value = 0.009, OR 2.178 95% CI 1.208-3.925), and in moderate-severe (28.3%, χ^2^ p-value = 4.514*10^-4^, OR 3.386 95% CI 1.668-6.877) *vs.* the reference set (10.5%). Similarly, the allelic counts of IL4-RP2*183 were significantly higher in moderate-severe *vs.* the reference set (26.7% *vs.* 10.5%; χ^2^ p-value = 0.001, OR 3.115 95% CI 1.521-6.379).

### SNP association to FEV_1_ and FVC

Table 4 shows the association analysis of SNP genotypes with the FEV_1_ and FVC indexes. Significant differences were found for mean FEV_1_ distribution among IL4-590*C/T genotypes (F = 3.982, p-value = 0.024), with IL4-590*CT/TT patients exhibiting a significantly lower FEV_1_ values when compared to IL4-590*CC homozygotes patients (t = 2.797, p-value = 0.006). Similarly, a significantly lower FEV_1_ index was found amongst patients with IL4-RP2*183253/183183 when compared to IL4-RP2*253253 homozygotes (t = 2.974, p-value = 0.004).

**Table 4:** **Mean FEV**_**1**_ **and FVC indexes across SNP genotypes**

SNP genotypes		Lung function indexes	
		FEV _1_ (mean +/-s.d.)	FVC (mean+/-s.d.)
n		98	98
*IL13-c.144 G/A*	GG	98.42 + -13.95	96.33 + -11.88
	GA	93.44 + -16.58	92.15 + -14.79
	AA	92.50 + -7.78	89.50 + -12.02
*IL4-590 C/T*	CC	99.92 + -14.28	96.87 + -11.28
	CT	91.69 + -14.41	92.13 + -14.79
	TT	90.00 + -12.00	87.67 + 16.04
**F p-value**		**0.024**	
**Dominant model CT/TT vs CC**		**0.006**	
*IL4-RP2 253/183*	253253	99.75 + -14.15	97.07 + -11.34
	183253	90.59 + -14.52	90.74 + -14.89
	183183	90.00 + -12.00	87.67 + -16.04
**F p-value**		**0.015**	
**Dominant model 183253/183183 vs 253253**		**0.004**	
*ADRB2-c.16 A/G*	AA	95.16 + -19.79	91.74 + -16.20
	AG	95.91 + -12.83	94.91 + -11.95
	GG	99.47 + -13.94	97.19 + -11.62
*ADAM33-V4 C/G*	GG	99.00 + -0 ^c^	98.00 + -0^c^
	CG	94.65 + -15.66	94.26 + -10.19
	CC	97.61 + -14.53	95.24 + -13.61
*ADAM33-S1c.710 G/A*	GG	97.13 + -14.81	95.22 + -13.05
	GA	94.29 + -13.96	92.71 + -8.92
	AA	-	-
*GSDML-236 C/T*	CC	99.13 + -13.20	94.25 + -12.07
	CT	96.30 + -17.48	94.13 + -14.34
	TT	96.75 + -11.34	96.56 + -11.04
*STAT6-21 C/T*	CC	97.34 + -15.74	95.00 + -14.28
	CT	96.49 + -13.52	94.24 + -11.73
	TT	96.92 + -15.73	97.69 + -11.01

^c^ n = 1.

A Shapiro-Wilkoxon test showed that FEV_1_ is normally distributed (p > 0.05) while FVC is not (p = 0.008). Thus, ANOVA was used for FEV_1_ analysis while a Kruskal-Wallis test was used for FVC.

### SNP association to SPT

Despite not associated to SPT positivity, some of the studied SNPs relate to the sensitization degree (Table 5). ADAM33-V4*CC relates to house dust mite (*Dermatophagoides pteronyssinus* χ^2^ p-value = 0.016, OR 1.325, 95% CI 1.070-1.641 and *Blomia tropicalis* χ^2^ p-value = 0.047, OR 1.355, 95% CI 1.131-1.622). STAT6-21*TT is associated to the degree of sensitization for storage mite (χ^2^ p-value = 0.003, OR 7.778, 95% CI 1.682- 35.962).

**Table 5:** **Skin prick tests positivity across SNP genotypes**

*Allergen panel*			SNP genotypes				
			*ADAM33-V4 C/G*				
		n	GG	CG	CC	p-value	OR (95% CI)
*Dermatophagoides pteronyssinus*	(-)	23	0.043	0.348	0.609		
	(+)	74	0	0.203	0.797		
	< 10 mm	44	0	0.295	0.705		
	≥ 10 mm	30	0	0.067	0.933	**0.016**	**1.325 (1.070-1.641)**
*Blomia tropicalis*	(-)	43	0.023	0.279	0.698		
	(+)	54	0	0.204	0.796		
	< 10 mm	42	0	0.262	0.738		
	≥ 10 mm	12	0	0	1.00	**0.047**	**1.355 (1.131-1.622)**
			*STAT6-21 C/T*				
			CC	CT	TT		
*Storage mites*	(-)	53	0.396	0.472	0.132		
	(+)	44	0.500	0.364	0.236		
	< 10 mm	35	0.514	0.429	0.057		
	≥ 10 mm	9	0.444	0.111	0.444	0.003*	**7.778 (1.682-35.962)**

Only significant associations are reported, in bold.

Storage mites: *Lepidoglyphus destructor*, *Glycifagus domesticus*, *Acarus siro*, *Euroglyphus maynei* and *Tyrophagus putrescentiae* allergens, classified as ≥ 10 mm if at least one test resulted in wheal diameter ≥ 10 mm.*Recessive model.

### SNP haplotypes and epistasis association to asthma risk

Table 6 shows the haplotype frequencies for SNPs at the 5q31 and 20p13 chromosomal regions. At the 5q31, we highlight IL4-590/IL4-RP2* T183 frequency, which is significantly lower in the reference set (8.8%) than overall asthma (16.8%, p-value = 0.019), persistent asthma (18.2%, p-value = 0.011), and moderate-severe asthma (26.7%, p-value = 3×10^-4^). In addition, this same haplotype was more common in moderate-severe *vs.* mild asthma (26.7% *vs*. 12.5%, p-value = 0.029). In fact, every haplotype found to associate with the risk of asthma includes either *IL4-590 C/T* or *IL4-RP2183/253* or both. Compared to the reference set, the most significant combinations are i) overall asthma IL13c.144/IL4-590*GT (p-value = 0.018); ii) persistent asthma IL13c.144/IL4-590*GT (p-value = 0.010) and *iii)* moderate-severe asthma IL4-590/IL4-RP2*T183 (p-value = 3x10^-4^). IL13c.144/IL4-RP2*A183 is also more frequently found in moderate-severe asthma than in mild asthma (p-value = 0.017).

**Table 6:** **SNP haplotype frequencies in the Madeira reference set and the asthmatics study population**

Haplotype block		Study sample set					
		Madeira reference set	Overall asthma	Intermittent asthma	Persistent asthma	Mild	Moderate-severe
		105^a^	98	24	74	44	30
*IL4-590/ IL4-RP2*	T183	0.088	**0.168 (0.019)**	0.125	**0.182 (0.011)**	0.125	**0.267 (3x10** ^**-4**^ /0.029*)
	C253	0.894	0.806	0.833	0.797	0.852	0.717 (0.004/0.044*)
*IL4-590 /ADRB2-c.16*	TA	0.018	**0.058 (0.037)**	0.064	**0.055 (0.048)**	0.031	**0.085 (0.009)**
	TG	0.092	0.136	0.102	0.148	0.117	**0.199 (0.023)**
*IL4-RP2/ADRB2-c.16*	183A	0.025	0.057	0.059	0.056	0.032	**0.089 (0.026)**
	183G	0.084	0.111	0.066	0.127	0.093	**0.178 (0.037)**
*IL13-c.144/ IL4-590*	GT	0.064	**0.134 (0.018)**	0.098	**0.146 (0.010)**	0.128	**0.166 (0.010)**
	AT	0.046	0.060	0.068	0.057	0.020	0.110 (0.020*)
*IL13-c.144/ IL4-RP2*	G183	0.059	0.106	0.052	**0.124 (0.033)**	0.105	**0.147 (0.022)**
	A183	0.050	0.063	0.073	0.059	0.021	0.114 (0.017*)
*IL13-c.144/ IL4-590/ IL4-RP2*	GT183	0.051	**0.107 (0.041)**	0.054	**0.125 (0.016)**	0.105	**0.148 (0.009)**
	AT183	0.040	0.062	0.071	0.058	0.020	0.119 (0.024/0.018*)
*IL4-590/ IL4-RP2/ ADRB2-c.16*	T183A	0.016	**0.055 (0.038)**	0.052	**0.053 (0.045)**	0.030	**0.082 (0.008)**
	T183G	0.073	0.113	0.067	0.129	0.095	**0.184 (0.013)**

Haplotypes denoting significant differences are shown in bold, with respective p-values within parentheses. Significant p-values, using Madeira reference set as a control, are shown within parentheses; * significant p-values for comparison between mild persistent and moderate-severe persistent.

Epistatic interactions between pairs of SNPs between all asthma subgroups were tested, revealing significant epistasis for *loci IL4-590* and *STAT6-21* in moderate-to-severe compared to intermittent asthma (p-value = 0.044) and *IL13-c.144* and *ADAM33-V4* between persistent asthma and the Madeira reference set (p-value = 0.035).

### TDT association analysis

The TDT analysis in Table 7 suggests that the ADAM33-V4*C allele is significantly over transmitted to offsprings in overall asthma (ratio 27/15 of transmitted/non-transmitted; McNemar χ^2^ 3.429, p-value = 0.044, OR 1.800, 95% CI 0.956-3.384), in persistent asthma (ratio 22/9 of transmitted/non-transmitted; McNemar χ^2^ 5.452, p-value = 0.015 OR 2.444, 95% CI 1.126-5.309), and in mild asthma (ratio 16/4 of transmitted/non-transmitted; McNemar χ^2^ 7.200 p-value = 0.006 OR 4.000, 95% CI 1.337-11.965). IL4-590*T and IL4-RP2*183 alleles were found to be significantly over transmitted only amongst moderate-severe offspring asthma (ratio 15/6 of transmitted/non-transmitted; McNemar χ^2^ 3.857, p-value = 0.039 OR 2.500, 95% CI 0.970-6.443 and ratio 14/4 of transmitted/non-transmitted; McNemar χ^2^ 5.556, p-value = 0.015 OR 3.500, 95% CI 1.152-10.663, respectively).

**Table 7:** **TDT analysis in family trios for SNP association with asthma**

SNP allele	Valid trios (n)	TDT-test value	TDT p-value	OR (95% CI)
**Overall asthma**				
ADAM33-V4*C	41	3.429	**0.044**	1.800 (0.956-3.384)
**Persistent asthma**				
ADAM33-V4*C	30	5.452	**0.015**	**2.444 (1.126-5.309)**
**Mild asthma**				
ADAM33-V4*C	19	7.200	**0.006**	**4.000 (1.337-11.965)**
**Moderate-severe asthma**				
IL4-590*T	19	3.857	**0.039**	2.500 (0.970-6.443)
IL4-RP2*183	17	5.556	**0.015**	**3.500 (1.152-10.633)**

Only significant associations are reported, in bold.

## Discussion

In the asthmatic patients studied, 24.5% are classified as intermittent asthmatics while the remaining are persistent asthmatics (of which 40.5% show moderate-severe forms of the disease). The significant differences we observed for FEV_1_ and FVC indexes denote these as reliable indicators for disease severity, as previous stated by a number of studies [48–50].

SNP *IL4-590 C/T* has been repeatedly associated to asthma in several population backgrounds [17–22] and the results of this study in the Madeira island population are no exception (previously reported by our research team [7]. Our findings show that IL4-590*CT/TT genotypes associate to a 2.2-fold risk for asthma (2.4-fold for overall persistent asthma and 4-fold for moderate-severe persistent asthma) while IL4-590*T allele represents a 2-fold risk for asthma (2.2-fold for persistent asthma and 3.4-fold for moderate-severe asthma), compared to individuals harbouring other *IL4-590 C/T* genotypes or allele. Our findings also indicate that IL4-RP2*253183/183183 genotypes translate into a 2-fold increased risk for asthma (2.3-fold and 2.9-fold respectively for persistent and moderate-severe persistent asthma), in particular IL4-RP2*183 allele (3.1-fold risk for moderate-severe persistent asthma). This SNP has been implicated in childhood asthma in a Chinese population, where authors suggest a link between the allele and higher expression of IL4 in asthma [24]. The findings for *IL4* SNPs are further corroborated by the association of the risk genotypes to worse lung function, namely lower FEV_1_ index. Genotype IL4-590*TT has been previously associated with lower FEV_1_ index, used as a measure for asthma severity, in a population of white asthmatic subjects [51].

IL4 is a key cytokine in IgE production via Th2 pathway [52], which has been previously shown to play an important role in asthma pathogenesis [53]. Its serous levels are significantly higher in steroid-naive asthmatic children and severe forms of asthma when compared to healthy control groups [54, 55]. From a functional perspective, it is not surprising that *IL4* SNPs may regulate its transcription rates: allele IL4-590*T allows an extra binding site for the nuclear factor of activated T-cells (NFAT) at *IL4* promoter, leading to a 3-fold higher transcription rate [23]; haplotypes with IL4-RP2*183 and SNPs within *IL4* promoter account for high IL4 expression [56].

The case–control associations of IL4-590*T and IL4-RP2*183 to moderate-severe persistent asthma are further corroborated by TDT analysis (2.5 and 3.5-fold increased risk respectively). TDT is a family-based test robust against population stratification [57, 58] defined as the presence of multiple subgroups with distinct allele frequencies within a population [59]. Although stratification is mostly noticed in recent history of admixture [60], we cannot disregard the considerable sub-Saharan and northern African proportion in the Madeira genetic background, due to the North Atlantic slavetrade on the 16^th^-19^th^ centuries and previously confirmed by Y-chromosome [61] and mtDNA studies [62].

Previous studies have related ADRB2-c.16*GG to asthma onset and severity [25, 26]. Conversely, our data suggest heterozygous ADRB2-c.16*AG as 3.5-fold risk genotype for moderate-severe asthma. In the absence of functional studies, we can only hypothesize that heterodimeric ADRB2 receptors on the bronchial smooth muscle may be less stable or bind to a more restricted scope of products than homodimeric receptors, therefore enhancing more severe forms of asthma. However, the high heterozygosity for *ADRB2-c.16* is found not only in patient’s subgroups but also in the Madeira reference set and other population sets [63–65]. Even though it deserves more attention, this may indicate heterozygote advantage for this SNP or other in linkage disequilibrium, in a metabolic pathway that may or may not relate to asthma pathogenesis. Other examples of heterozygote advantage in *ADRB2* exist, such as ADRB2-c.27*CG that is protective against lung function decline [66] and accounts for a decreased risk of asthma [67].

In our data, GSDML-236*TT genotype is found to be a protective for asthma severity, with a 4-fold significantly lower frequency in moderate-severe asthma, as opposed to mild. *GSDML-236C/T* polymorphism has been related to asthma predisposition, severity and/or asthma-related phenotypes in a number of studies [31–39]. However, our results are in contrast to certain previous studies, which suggest this genotype is a genetic risk factor for the disease severity [32, 35, 37, 38]. The *GSDML* encodes for gasdermin B protein expressed in epithelial barrier function and skin differentiation, influencing the expression of the neighbouring gene *ORMDL3* and thus contributing to asthma susceptibility [34].

The case–control association in our dataset does not support the influence of *ADAM33-V4C/G* or *ADAM33-S1c.710G/A* in asthma, contrary to some previous work [27–30] and consistent with other studies [68]. ADAM33-V4*C allele was nevertheless marginally over transmitted to Madeira asthmatics, as suggested by TDT analysis (1.8-fold risk for overall asthma, 2.4-fold risk for persistent asthma and 4-fold risk for mild asthma). A similar trend of association was seen in Caucasian children with asthma and bronchial responsiveness (p-value = 0.0959) [27]. Located at 3’-UTR, *ADAM33-V4C/G* SNP may influence the expression of its encoded desintegrin and metallopeptidase domain 33 in airway smooth muscle and lung fibroblasts [27, 69, 70] thus playing a role on airway remodeling and bronchial responsiveness [27, 71]. In addition, our study associates ADAM33-V4*CC to a wheal size ≥ 10 mm both in *Dermatophagoides pteronyssinus* and *Blomia tropicalis*, in accordance with a previous study relating ADAM33-V4*CC to high levels of IgE against *Blomia tropicalis* (Z score: 2.089, p = 0.03) [72].

No significant association with the disease risk was detected for *STAT6-21C/T,* similar to the analysis in [73] but contrary to other studies [19, 40–44]. However, STAT6-21*TT is overrepresented in asthmatics with a ≥10 mm wheal size reaction to storage mites and shows a significant epistatic interaction with *IL4-590C/T* between moderate-severe persistent and intermittent asthma. The enhanced allergic response is not unexpected since STAT6 is a signal transducer and activator of transcription enrolled in the regulation of IgE expression [40, 52, 74] whose STAT6-21*T allele promotes higher levels of IgE [19].

Finally, the haplotype analysis was significant for SNPs at 5q31, namely IL4-590*T and IL4-RP2*183 in overall asthma, persistent asthma and moderate-severe persistent asthma. Haplotypes of IL4-RP2*183 and SNPs within the promoter of *IL4* have been found to account for high IL4 expression [56]. Our results also indicate the synergistic effect of IL13c.144*G and ADRB2*G with either IL4-590*T or IL4-RP2*183 or both for the onset of asthma persistent phenotypes. Nevertheless, their combination equals or slightly lowers the statistical significance of IL4-590/IL4-RP2*T183 haplotype in the same classes.

## Conclusion

In conclusion, this study has replicated previously reported genetic associations and brought light to new associations of polymorphisms, individually or in haplotype, to asthma susceptibility, persistence and/or severity in the Madeira island population. *IL4-590C/T* and *IL4-RP2253/183* seem to play an important role in the development of the disease, especially its moderate-severe persistent forms, as demonstrated by allele and haplotype case–control tests, TDT analysis, and significantly poorer FEV_1_ index. GSDML*CT/TT and ADRB2c.16*AG are found to predict asthma severity in case–control association, respectively as risk and protective factors for moderate-severe forms. ADAM33-V4*C and STAT-21*TT seem to enhance the allergic response to *Dermatophagoides pteronyssinus* and *Blomia tropicalis* and to storages mites, respectively, and the first is further highlighted by TDT analysis as a risk factor for mild persistent asthma. The combined genotyping of *IL4-590C/T, IL4-RP2-253/183, GSDML-236C/T* and *ADAM33-V4C/G* could be of clinical relevance in ascertaining the risk of asthma onset and/or its persistence and severity, thus helping to define appropriate strategies for asthma healthcare in this island.

## Methods

### Participants

A sample of 98 atopic asthmatics, aged between 6 and 25 years old, and their parents (fathers n = 88 and mothers n = 96) were recruited at the Immunoalergology Unit, Dr. Nélio Mendonça Hospital, in Funchal, Madeira. For all participants, the last three generations of ancestors were born and lived in Madeira island. The diagnosis of asthma was based on clinical criteria associated with lung function and the SPT. For the epidemiologic characterization we deployed the ISAAC questionnaire to the asthmatic population and their parents. Following the GINA guidelines [75], pattern and frequency of symptoms associated with lung function were used to classify asthma into intermittent (n = 24) and persistent (n = 74), and the severity in persistent asthma was further classified as mild (n = 44), moderate (n = 26) and severe (n = 4). Given the small number of patients, the moderate and severe subgroups were merged. Asthma induced by exercise and associated to aspirin was excluded.

### Ethics statement

The study had the approval of the Ethics Committee of Dr. Nélio Mendonça Hospital and the informed consent of the participants.

### Allergy profile

The SPT was tested for an optimized battery with the most common aeroallergens in this island: house dust mite (*Dermatophagoides pteronyssinus, Blomia tropicalis*)*;* storage mite (*Lepidoglyphus destructor, Glycyphagus domesticus, Acarus siro, Euroglyphus maynei, Tyrophagus putrescentiae*)*;* Mix fungi (Fungus I*,* Fungus II)*;* indoor fungi (*Aspergillus fumigatus, Mucor sp.*) and cat and dog. STP results were interpreted according to mean wheal size: positive ≥3 mm, sensitization degree ≤10 mm or ≥10 mm.

### DNA extraction and genotyping

Patients’ blood samples were collected by venipuncture in 8 ml EDTA blood collection tubes and their DNA was extracted by salting-out method (adapted from [76]). Their parents contributed with saliva samples, collected in Oragene•DNA (OG-250) kits (Oragene). The DNA extraction followed the manufacturer’s instructions.

A sample of 110 unrelated individuals, whose ancestry was of local origin for the last three generations, was obtained from the Blood Sample Bank of Human Genetics Laboratory (LGH) and their DNA extraction was performed by a standard phenol-chloroform protocol (adapted from [77]). These constitute the Madeira population reference sample set, although their disease status is unknown. The reference sample set was not matched to the patient’s group to uphold a less biased study design. Each SNP (Table 1) was analysed by TaqMan real-time PCR (Applied Biosystems) according to the manufacturer’s instructions, in a 7300 System SDS Software v1.4.

### Statistical analysis

The statistical analysis was performed using SPSS (IBM SPSS Statistics, version 19.0.0), except for the Hardy-Weinberg Equilibrium (HWE) for each polymorphism, determined in ARLEQUIN version 3.11 [78]. Gender and SPT data among subgroups of asthma persistence and severity were analysed using Fisher’s exact test, to account for the small sample size.

Following a Shapiro-Wilkoxon test of normality, the association analysis of FEV_1_ to asthma persistence and severity was performed using a t-test (normal distribution), while a Mann–Whitney test was used for FVC (due to its non-normal distribution). The analysis of FEV_1_ and FVC indexes among SNP genotypes was tested using ANOVA (with Bonferroni post-hoc correction) and Kruskal-Wallis test, respectively.

The association of SNP genotypes and alleles to asthma susceptibility and severity, for multiple genetic models, were assessed by χ^2^ (or Fisher’s exact tests where appropriate) and corresponding odds ratio (OR) determined with a 95% CI. Haplotypes were generated with Haploview version 4.2 [79] and their association with the disease was analysed with the hap-assoc option in PLINK [80]. Epistatic interactions between pairs of SNPs were assessed with the PLINK option --epistasis --epi1 1. Permutation procedures were used to obtain significance levels empirically and results were interpreted at a significance level set for p < 0.05.

The transmission disequilibrium test (TDT) was determined through a McNemar test [81], based on data from heterozygous parents and the frequency of allelic transmission to affected offspring. The corresponding p-value (considered at a 0.05 significance level) and OR (95% CI) were determined.

To reduce the likelihood of commiting a Type I error due to multiple testing, the Benjamini & Hochberg procedure [82] was adopted for each set of results (presented in distinct tables) to control the false discovery rate set at Q = 0.20.

## Electronic supplementary material

Additional file 1: **Skin prick tests positivity across asthma persistence and severity subgroups.** The table describes the qualitative (positive *vs.* negative) and quantitative (<10 mm vs. ≥ 10 mm) reaction to common allergens in Madeira island (*Dermatophagoides pteronyssinus, Blomia tropicalis,* Storage mites, Mix fungi, Indoor fungi, cat and dog) across overall asthma and asthma severity categories (intermittent, persistent, mild and moderate-severe). (DOCX 106 KB)

Additional file 2: **SNP allelic frequencies for the Madeira reference set and asthmatics study population.** The table describes the allelic frequencies for SNPs *IL4-590, IL4-RP2, IL13-c.144, ADRB2-c.16, ADAM33-V4, ADAM33-S1c.710, GSDML-236, STAT6-21*) across the reference group, overall asthma and asthma severity categories (intermittent, persistent, mild and moderate-severe). (DOCX 96 KB)

## Abbreviations

ADAM33: A Disintegrin and Metalloprotease 33
ADRB2: Beta-2-adrenergic receptor
FEV_1_: Forced Expiratory Volume in 1 second
FVC: Forced Vital Capacity
GINA: Global Initiative for Asthma
GSDML: Gasdermin-like
IL4: Interleukin 4
IL13: Interleukin 13
SNPs: Single Nucleotide Polymorphisms
SPT: Skin Prick Tests
STAT6: Signal Transducer and Activator of Transcription 6 (STAT6)
TDT: Transmission Disequilibrium Test.
