# Supplementary material for: Genetic polymorphisms and asthma: findings from a case–control study in the Madeira island population
Source: Biol Res. 2014 Sep 4;47(1):40. doi: 10.1186/0717-6287-47-40 (PMC4167518; doi:10.1186/0717-6287-47-40)
Supplement: Supplementary file 1 — Additional file 1: Skin prick tests positivity across asthma persistence and severity subgroups. The table describes the qualitative (positive vs. negative) and quantitative (<10 mm vs. ≥ 10 mm) reaction to common allergens in Madeira island (Dermatophagoides pteronyssinus, Blomia tropicalis, Storage mites, Mix fungi, Indoor fungi, cat and dog) across overall asthma and asthma severity categories (intermittent, persistent, mild and moderate-severe). (DOCX 106 KB) [file 40659_2014_37_MOESM1_ESM.docx]

Additional file 1. *Skin prick tests positivity across asthma persistence and severity subgroups.*

| **Allergen panel** | **Study sample set** | | | | |
| --- | --- | --- | --- | --- | --- |
|  | Overall asthma | Intermittent asthma | Persistent asthma | Mild | Moderate-Severe |
|  | n (%) | n (%) | n (%) | n (%) | n (%) |
|  | 97 | 23 | 74 | 44 | 30 |
| *Dermatophagoides pteronyssinus* | |  |  |  |  |
| (-) | 23 (23.7) | 7 (30.4) | 16 (21.6) | 8 (18.2) | 8 (26.7) |
| (+) | 74 (76.3) | 16 (69.6) | 58 (78.4) | 36 (81.8) | 22 (73.3) |
| < 10 mm | 44 (59.5) | 10 (62.5) | 34 (58.6) | 22 (61.1) | 12 (54.5) |
| ≥ 10 mm | 30 (40.5) | 6 (37.5) | 24 (41.4) | 14 (38.9) | 10 (45.5) |
| *Blomia tropicalis* |  |  |  |  |  |
| (-) | 43 (44.3) | 9 (39.1) | 34 (45.9) | 18 (40.9) | 16 (53.3) |
| (+) | 54 (55.7) | 14 (60.9) | 40 (54.1) | 26 (59.1) | 14 (46.7) |
| < 10 mm | 42 (77.8) | 11 (78.6) | 31 (77.5) | 20 (76.9) | 11 (78.6) |
| ≥ 10 mm | 12 (22.2) | 3 (21.4) | 9 (22.5) | 6 (23.1) | 3 (21.4) |
| Storage mites |  |  |  |  |  |
| (-) | 53 (54.6) | 13 (56.5) | 40 (54.1) | 22 (50.0) | 18 (60.0) |
| (+) | 44 (45.4) | 10 (43.5) | 34 (45.9) | 22 (50.0) | 12 (40.0) |
| < 10 mm | 35 (79.5) | 9 (90.0) | 26 (76.5) | 18 (81.8) | 8 (66.7) |
| ≥ 10 mm | 9 (20.5) | 1 (10.0) | 8 (23.5) | 4 (18.2) | 4 (33.3) |
| Mix fungi |  |  |  |  |  |
| (-) | 88 (90.7) | 22 (95.7) | 66 (89.2) | 39 (88.6) | 27 (90.0) |
| (+) | 9 (9.3) | 1 (4.3) | 8 (10.8) | 5 (11.4) | 3 (10.0) |
| Indoor fungi |  |  |  |  |  |
| (-) | 82 (84.5) | 19 (826) | 63 (85.1) | 37 (84.1) | 26 (86.7) |
| (+) | 15 (15.5) | 4 (17.4) | 11 (14.9) | 7 (15.9) | 4 (13.3) |
| Cat |  |  |  |  |  |
| (-) | 80 (82.5) | 22 (95.7) | 58 (78.4) | 34 (77.3) | 24 (80.0) |
| (+) | 17 (17.5) | 1 (4.3) | 16 (21.6) | 10 (22.7) | 6 (20.0) |
| Dog |  |  |  |  |  |
| (-) | 78 (80.4) | 22 (95.7) | 56 (75.7) | 33 (75.0) | 23 (76.7) |
| (+) | 19 (19.6) | 1 (4.3) | 18 (24.3) | 11 (25.0) | 7 (23.3) |
| **p-value** |  | **0.038* ^a^** |  | **0.046** ^a^** |  |
| **OR  (95%CI)** |  | **7.071**  **(0.889-56.218)** |  | **7.333**  **(0.883-60.912)** |  |

Storage mites: *Lepidoglyphus* *destructor*, *Glycifagus domesticus*, *Acarus siro*, *Euroglyphus maynei* and *Tyrophagus putrescentiae* allergens, classified as ≥ 10 mm if at least one test resulted in wheal diameter ≥ 10 mm; Mix fungi: Fungus I and Fungus II allergens, classified as positive if at least one test resulted positive; Indoor fungi: *Aspergillus fumigatus* and *Mucor sp*., classified as positive if at least one test resulted positive. Allergens were classified as: (+) if at least one test resulted positive wheal diameter ≥ 3 mm; ≥ 10 mm if at least one test resulted in wheal diameter ≥ 10 mm. *between persistent and intermittent asthma; **between mild and intermittent asthma, by using Fisher’s exact test. ^a^ Discarded after correction for false discovery rate, according to the Benjamini & Hochberg (1995) procedure at Q=0.20
