# Supplementary material for: Genetic polymorphisms and asthma: findings from a case–control study in the Madeira island population
Source: Biol Res. 2014 Sep 4;47(1):40. doi: 10.1186/0717-6287-47-40 (PMC4167518; doi:10.1186/0717-6287-47-40)
Supplement: Supplementary file 2 — Additional file 2: SNP allelic frequencies for the Madeira reference set and asthmatics study population. The table describes the allelic frequencies for SNPs IL4-590, IL4-RP2, IL13-c.144, ADRB2-c.16, ADAM33-V4, ADAM33-S1c.710, GSDML-236, STAT6-21) across the reference group, overall asthma and asthma severity categories (intermittent, persistent, mild and moderate-severe). (DOCX 96 KB) [file 40659_2014_37_MOESM2_ESM.docx]

Additional file 2. *SNP allelic frequencies for the Madeira reference set and asthmatics study population.*

| **SNP alleles** | **Study sample set** | | | | | |
| --- | --- | --- | --- | --- | --- | --- |
|  | Madeira reference set | Overall asthma | Intermittent asthma | Persistent asthma | Mild | Moderate-Severe |
|  | 105^a^ | 98 | 24 | 74 | 44 | 30 |
| *IL4-590*C* | 0.895 | 0.806 | 0.833 | 0.797 | 0.852 | 0.717 |
| *IL4-590*T* | 0.105 | 0.194 | 0.167 | 0.203 | 0.148 | 0.283 |
| **p-value** |  | **0.010** |  | **0.009** |  | **4.514*10^-4^** |
| **OR (95%CI)** |  | **2.060 (1.178 -3.601)** |  | **2.178 (1.208 -3.925)** |  | **3.386 (1.668- 6.877)** |
| **p-value** |  |  |  |  |  | **0.044* ^c^** |
| **OR (95%CI)** |  |  |  |  |  | **2.281 (1.011-5.146)** |
| *IL4-RP2*253* | 0.895 | 0.832 | 0.875 | 0.818 | 0.875 | 0.733 |
| *IL4-RP2*183* | 0.105 | 0.168 | 0.125 | 0.182 | 0.125 | 0.267 |
| **p-value** |  |  |  | **0.033 ^c^** |  | **0.001** |
| **OR (95%CI)** |  |  |  | **1.911 (1.048-3.484)** |  | **3.115 (1.521-6.379)** |
| **p-value** |  |  |  |  |  | **0.028* ^c^** |
| **OR (95%CI)** |  |  |  |  |  | **2.546 (1.085-5.970)** |
| *IL13-c.144*G* | 0.829 | 0.842 | 0.792 | 0.858 | 0.875 | 0.833 |
| *IL13-c.144*A* | 0.171 | 0.158 | 0.208 | 0.142 | 0.125 | 0.167 |
| *ADRB2-c.16*A* | 0.410 | 0.434 | 0.479 | 0.419 | 0.432 | 0.400 |
| *ADRB2-c.16*G* | 0.590 | 0.566 | 0.521 | 0.581 | 0.568 | 0.600 |
| *ADAM33-V4*G* | 0.133 | 0.128 | 0.146 | 0.122 | 0.114 | 0.133 |
| *ADAM33-V4*C* | 0.867 | 0.872 | 0.854 | 0.878 | 0.886 | 0.867 |
| *ADAM33-S1c.710*G* | 0.952 | 0.964 | 0.958 | 0.966 | 0.966 | 0.967 |
| *ADAM33-S1c.710*A* | 0.048 | 0.036 | 0.042 | 0.034 | 0.034 | 0.033 |
| *GSDML-236*C* | 0.405 | 0.398 | 0.417 | 0.392 | 0.318 | 0.500 |
| *GSDML-236*T* | 0.595 | 0.602 | 0.583 | 0.608 | 0.682 | 0.500 |
| **p-value** |  |  |  |  |  | **0.026* ^c^** |
| **OR (95%CI)** |  |  |  |  |  | **0.467 (0.237-0.918)** |
| *STAT6-21*C* | 0.671 | 0.658 | 0.646 | 0.662 | 0.670 | 0.650 |
| *STAT6-21*T* ^b^ | 0.329 | 0.342 | 0.354 | 0.338 | 0.329 | 0.350 |

^a^ Except for *IL4-590* and *IL4-RP2* (n=110);

^b^ Polymorphism not in HWE for mild asthma (p-value=0.038).

χ^2^significant p-values and OR (95%CI), using Madeira reference set as a control, are shown in bold.

*Significant p-values and OR (95%CI) for comparison between mild persistent and moderate-severe persistent asthma.

^c^ Discarded after correction for false discovery rate, according to the Benjamini & Hochberg (1995) procedure at Q=0.20.
